# Adaptive slice‐specific z‐shimming for 2D spoiled gradient‐echo sequences

**DOI:** 10.1002/mrm.28468

**Published:** 2020-09-10

**Authors:** Martin Soellradl, Johannes Strasser, Andreas Lesch, Rudolf Stollberger, Stefan Ropele, Christian Langkammer

**Affiliations:** ^1^ Department of Neurology Medical University of Graz Graz Austria; ^2^ Institute of Medical Engineering Graz University of Technology Graz Austria

**Keywords:** field inhomogeneities, gradient‐echo, $R_{2}^{*}$ relaxometry, $T_{2}^{*}$ relaxometry, z‐shim

## Abstract

**Purpose:**

To reduce the misbalance between compensation gradients and macroscopic field gradients, we introduce an adaptive slice‐specific z‐shimming approach for 2D spoiled multi‐echo gradient‐echoe sequences in combination with modeling of the signal decay.

**Methods:**

Macroscopic field gradients were estimated for each slice from a fast prescan (15 seconds) and then used to calculate slice‐specific compensation moments along the echo train. The coverage of the compensated field gradients was increased by applying three positive and three negative moments. With a forward model, which considered the effect of the slice profile, the z‐shim moment, and the field gradient, $R_{2}^{*}$ maps were estimated. The method was evaluated in phantom and in vivo measurements at 3 T and compared with a spoiled multi‐echo gradient‐echo and a global z‐shimming approach without slice‐specific compensation.

**Results:**

The proposed method yielded higher SNR in $R_{2}^{*}$ maps due to a broader range of compensated macroscopic field gradients compared with global z‐shimming. In global white matter, the mean interquartile range, proxy for SNR, could be decreased to 3.06 s^−1^ with the proposed approach, compared with 3.37 s^−1^ for global z‐shimming and 3.52 s^−1^ for uncompensated multi‐echo gradient‐echo.

**Conclusion:**

Adaptive slice‐specific compensation gradients between echoes substantially improved the SNR of $R_{2}^{*}$ maps, and the signal could also be rephased in anatomical areas, where it has already been completely dephased.

## 1 INTRODUCTION

Magnetic resonance imaging sequences based on gradient‐echo (GRE) readout strategies play a major role in clinical routine, and because of their low specific absorption rate behavior, they are used increasingly at ultrahigh field strengths. Besides the morphological information provided by GRE images, the decay of the complex signal offers insights into the underlying tissue compartments and their susceptibilities.^1^, ^2^, ^3^, ^4^, ^5^, ^6^, ^7^, ^8^, ^9^, ^10^, ^11^ Over the entire physiological range, the effective transverse relaxation rate $R_{2}^{*}$ serves as a proxy for iron concentration and has been used to study inflammatory and degenerative diseases in the brain,^12^, ^13^ iron overload in the liver,^14^ and myocardial iron overload.^15^

However, obtaining quantitative $R_{2}^{*}$ values from gradient‐echoes is typically subject to quantification errors due to faster intravoxel dephasing caused by macroscopic field variations^16^ (such as near air/tissue interfaces). In 2D spoiled GRE imaging, dephasing is more pronounced along the slice direction because the slice thickness is usually much larger than the in‐plane resolution. Consequently, an effective approach to reduce signal dephasing is to decrease the slice thickness, which is accompanied by reduced SNR and prolonged acquisition time.^17^ Various postprocessing methods for reducing these dephasing effects by considering the slice profile and macroscopic field variations have been proposed,^18^, ^19^, ^20^, ^21^, ^22^, ^23^ but at very strong field gradients the correction of the fast signal decay might not be feasible.

Alternatively, z‐shimming approaches allow compensating signal dephasing due to a certain macroscopic field gradient $G_{z}$ by variation of the compensation gradient moment in the slice‐selective direction. Starting from the basic principle of changing the amplitude of the slice‐selective refocusing gradient demonstrated by Frahm et al,^24^ different methods have evolved. To minimize the effects of $G_{z}$ on $T_{2}^{\prime }$ quantification, Ordidge et al proposed the acquisition of images with different refocusing gradients.^25^ Similarly, the GRE slice excitation profile imaging method acquires several images with an equidistant spacing of compensation gradients to estimate the k‐space shift in z‐direction, which allows reconstruction of a magnitude image with minimal contribution of $G_{z}$.^26^ The method was applied to $R_{2}^{*}$ mapping,^27^ and by adding compensation gradients between the echo acquisitions, a more efficient sampling is possible.^28^ Nonetheless, a drawback of these approaches is that several additional images need to be acquired, which limits their application in clinical routine because of the prolonged scan time.

Similarly, Meng et al started with one strong compensation gradient before acquiring the echo train and successively rephased it with small opposed inter‐echo gradients.^29^ To reduce acquisition time, Wild et al proposed a single‐scan method by applying repetitively a triple of compensation gradients between echo acquisition of a spoiled multi‐echo GRE sequence (mGRE).^30^ However, all of these methods assume an ideal slice profile, which would give rise to a sinc‐shaped signal decay in the presence of $G_{z}$.^16^ Addressing variations of slice profile, Nam et al proposed a single‐scan z‐shim approach that includes the slice profile and compensates for a positive and negative $G_{z}$ by applying compensation gradients that are linearly scaled with TE.^31^ To avoid signal crushing of the first echoes, Lee et al started the z‐shim after the fifth echo for myelin water signal mapping.^32^

A common limitation of the aforementioned approaches is that the compensation gradients are fixed for the entire FOV (global z‐shim), and, consequently, a misbalance with the actual field gradient results in incomplete rephasing or even spoiling of the signal.

We therefore propose an adaptive slice‐specific z‐shimming approach to address spatial variations of $G_{z}$. The corresponding slice‐specific compensation gradients are estimated for each slice individually from a fast prescan. Additionally, a more effective z‐shim pattern is introduced, in which six $G_{z}$ values are successively compensated between echo acquisitions. By adapting a signal modeling approach for 2D spoiled mGRE sequences,^23^ we compare this novel approach, in terms of $R_{2}^{*}$ mapping, with a global z‐shim approach with linearly increasing moments^31^, ^32^ and a conventional mGRE sequence without z‐shim gradients. Furthermore, to highlight the importance of adequate signal modeling in the presence of $G_{z}$, $R_{2}^{*}$ is also estimated from the conventional mGRE data with a more widespread used mono‐exponential signal model.

## 2 METHODS

### 2.1 Signal modeling

Signal dephasing due to a field gradient $G_{z}$ can be compensated at a TE by applying a short compensation gradient with duration $t_{c}$ and amplitude $G_{c}$, which results in a compensation moment $m_{c}=G_{c}t_{c}=‐G_{z}TE$. In the case of a train of *k* compensation gradients, each with the amplitude $G_{c}\left[k\right]$ and identical $t_{c}$, the accumulative moment $M_{c}\left[i\right]$ for the *i*th echo at *TE*_*i*_ is given by:

(1) $$M_{c}\left[i\right]=\sum_{k=1}^{i}m_{c}\left[k\right]=\sum_{k=1}^{i}G_{c}\left[k\right]t_{c}={\bar{G}}_{c}\left[i\right]TE_{i}.$$

The sum of all applied compensation moments $m_{c}\left[k\right]$ until $TE_{i}$ is equal to a single theoretical mean compensation gradient ${\bar{G}}_{c}\left[i\right]$ applied over the entire duration $TE_{i}$. This allows us to superimpose $G_{z}$ and ${\bar{G}}_{c}\left[i\right]$ for signal modeling independent of the shape and duration of the applied compensation gradients. Assuming a mono‐exponential signal decay with $R_{2}^{*}$, the signal $S\left(TE_{i}\right)$ of the spoiled gradient echo is given by integrating the complex transverse magnetization ${\underline{M}}_{\mathrm{xy}}\left(z\right)$ weighted with the phase dispersion induced by both gradients along the z‐direction:

(2) $$\begin{array}{ccc} S\left(TE_{i}\right) & = & S_{0}e^{‐R_{2}^{*}TE_{i}}\int_{‐\infty }^{\infty }{\underline{M}}_{\mathrm{xy}}\left(z\right)e^{i\gamma \left(G_{z}+{\bar{G}}_{c}\left[i\right]\right)zTE_{i}}dz\\ & =S_{0}e^{‐R_{2}^{*}TE_{i}}F_{z‐shim}\left(TE_{i}\right) \end{array}$$

where $S_{0}$ describes the signal $S\left(TE=0\right)$, and $F_{z‐shim}\left(TE_{i}\right)$ summarizes the net effect of $G_{z}$ and ${\bar{G}}_{c}\left[i\right]$. In the case of small flip angles, the resulting signal decay is described by the pulse envelope of the RF excitation pulse.^20^ Otherwise, the integral in Equation 2 can be solved numerically, where ${\underline{M}}_{\mathrm{xy}}\left(z\right)$ is obtained by the numerical solution of the Bloch equations.^22^, ^23^

### 2.2 Sequence

**FIGURE 1:**
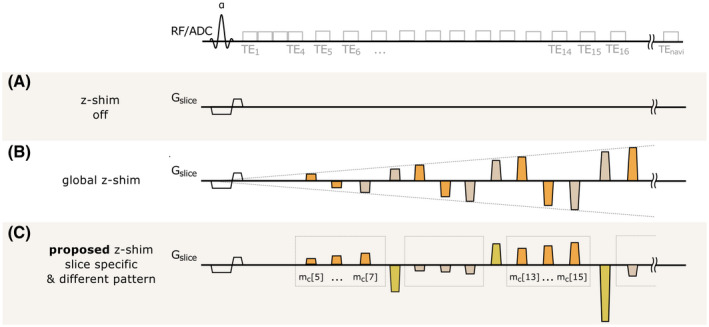
Schematic overview of the compared sequences. A, Spoiled multi‐echo gradient‐echo (mGRE) sequence without z‐shimming. B, In the global z‐shim approach, moments are applied through alternating pairs (same color) with a linear increase along TE. The first moment in each pair is calculated based on a single positive or negative ${\bar{G}}_{c}^{+/‐}$, and the second moment balances the compensation moment to acquire a gradient‐echo (GRE) image with zero net moment. C, The proposed slice‐specific approach, with ${\bar{G}}_{c}^{+/‐}\left[n\right]$ estimated from a prescan individually for each slice *n*. In addition, ${\bar{G}}_{c}^{+/‐}\left[n\right]$ is split up with factors $\left[\frac{1}{3},\frac{2}{3},\frac{3}{3}\right]{\bar{G}}_{c}^{+/‐}\left[n\right]$ (dashed boxes) followed by compensation of all moments. To correct for physiological fluctuations, a navigator echo is acquired at TE_navi_

Figure 1 shows a 2D spoiled mGRE sequence (Figure 1A) and a combination of the global z‐shim patterns proposed by Nam et al and Lee et al^31^, ^32^ (Figure 1B) along with the proposed slice‐specific pattern presented in this work (Figure 1C). In addition, Table 1 lists the corresponding compensation gradients ${\bar{G}}_{c}\left[i\right]$ for the z‐shim approaches for each echo.

**TABLE 1:** Mean compensation gradients ${\bar{G}}_{c}\left[i\right]$ (based on Equation (1), (2)) for the global and the proposed z‐shim approaches as a function of echo number *i*

Echo *i*		1	…	4	5	6	7	8	9	10	11	12	13	14
Global z‐shim	${\bar{G}}_{c}^{+}$, ${\bar{G}}_{c}^{‐}$	0	…	0	${\bar{G}}_{c}^{+}$	0	${\bar{G}}_{c}^{‐}$	0	${\bar{G}}_{c}^{+}$	0	${\bar{G}}_{c}^{‐}$	0	${\bar{G}}_{c}^{+}$	…
Proposed z‐shim	${\bar{G}}_{c}^{+}\left[n\right]\neq 0,$ ${\bar{G}}_{c}^{‐}\left[n\right]\neq 0$	0	…	0	$\frac{1}{3}{\bar{G}}_{c}^{+}\left[n\right]$	$\frac{2}{3}{\bar{G}}_{c}^{+}\left[n\right]$	$\frac{3}{3}{\bar{G}}_{c}^{+}\left[n\right]$	0	$\frac{1}{3}{\bar{G}}_{c}^{‐}\left[n\right]$	$\frac{2}{3}{\bar{G}}_{c}^{‐}\left[n\right]$	$\frac{3}{3}{\bar{G}}_{c}^{‐}\left[n\right]$	0	$\frac{1}{3}{\bar{G}}_{c}^{+}\left[n\right]$	…
	${\bar{G}}_{c}^{‐}\left[n\right]=0$	0	…	0	$\frac{1}{5}{\bar{G}}_{c}^{+}\left[n\right]$	$\frac{2}{5}{\bar{G}}_{c}^{+}\left[n\right]$	$\frac{3}{5}{\bar{G}}_{c}^{+}\left[n\right]$	$\frac{4}{5}{\bar{G}}_{c}^{+}\left[n\right]$	$\frac{5}{5}{\bar{G}}_{c}^{+}\left[n\right]$	0	$\frac{1}{5}{\bar{G}}_{c}^{+}\left[n\right]$	$\frac{2}{5}{\bar{G}}_{c}^{+}\left[n\right]$	$\frac{3}{5}{\bar{G}}_{c}^{+}\left[n\right]$	…
	${\bar{G}}_{c}^{+}\left[n\right]=0$	0	…	0	$\frac{1}{5}{\bar{G}}_{c}^{‐}\left[n\right]$	$\frac{2}{5}{\bar{G}}_{c}^{‐}\left[n\right]$	$\frac{3}{5}{\bar{G}}_{c}^{‐}\left[n\right]$	$\frac{4}{5}{\bar{G}}_{c}^{‐}\left[n\right]$	$\frac{5}{5}{\bar{G}}_{c}^{‐}\left[n\right]$	0	$\frac{1}{5}{\bar{G}}_{c}^{‐}\left[n\right]$	$\frac{2}{5}{\bar{G}}_{c}^{‐}\left[n\right]$	$\frac{3}{5}{\bar{G}}_{c}^{‐}\left[n\right]$	…

Note

Compensation moments in the global z‐shim approach are calculated from a single positive ${\bar{G}}_{c}^{+}$ and negative ${\bar{G}}_{c}^{‐}$, whereas with the proposed approach, slice‐specific values for ${\bar{G}}_{c}^{+}\left[n\right]$ and ${\bar{G}}_{c}^{‐}\left[n\right]$ are estimated for each slice *n*. To increase coverage of compensated field gradients, these values are divided into three fractions or five fractions in case of ${\bar{G}}_{c}^{+}\left[n\right]$ or ${\bar{G}}_{c}^{‐}\left[n\right]$ is being equal to zero.

The compensation moments for the global z‐shim method (Figure 1B) are calculated for a single positive ${\bar{G}}_{c}^{+}$ and negative ${\bar{G}}_{c}^{‐}$ value. The first applied gradient moment after the fourth echo ($m_{c}\left[5\right]={\bar{G}}_{c}^{+}TE_{5}=‐G_{z}^{‐}TE_{5}$) compensates for the effects of negative $G_{z}^{‐}$ followed by nulling the accumulative moment by inverting $m_{c}\left[5\right]$ ($m_{c}\left[6\right]=‐m_{c}\left[5\right]$). This step is repeated for a positive $G_{z}^{+}$ by applying a negative compensation moment ($m_{c}\left[7\right]={\bar{G}}_{c}^{‐}TE_{7}=‐G_{z}^{+}TE_{7}$). To avoid crushing of the signal in the first echoes, z‐shim gradients are not applied for the first echoes as proposed by Lee et al.^32^

Our work extends the compensation pattern in Figure 1B by two novel contributions. First, instead of using global ${\bar{G}}_{c}^{+/‐}$ for all slices, slice‐specific compensation gradients ${\bar{G}}_{c}^{+/‐}\left[n\right]$ are applied for each slice n. These ${\bar{G}}_{c}^{+/‐}\left[n\right]$ values are estimated from a field map measured with a fast prescan. Second, instead of a single ${\bar{G}}_{c}^{+}\left[n\right]$ and ${\bar{G}}_{c}^{‐}\left[n\right]$, the coverage of compensated $G_{z}^{+/‐}$ values is increased by a successive application of three positive and three negative compensation moments. Based on the estimated ${\bar{G}}_{c}^{+/‐}\left[n\right]$, the moments between echoes are scaled such that $\left[\frac{1}{3},\frac{2}{3},\frac{3}{3}\right]{\bar{G}}_{c}^{+/‐}\left[n\right]$ are compensated for three consecutive echoes, which is followed by a nulling of the total moment for the subsequent echo. To give an example, the moments $m_{c}\left[n,5\right]$ to $m_{c}\left[n,7\right]$ in the proposed pattern (Figure 1C) are calculated as follows, assuming equal echo spacing ΔTE:

(3) $$m_{c}\left[n,5\right]=\frac{1}{3}{\bar{G}}_{c}^{+}\left[n\right]TE_{5}$$

(4) $$m_{c}\left[n,6\right]={\bar{G}}_{c}^{+}\left[n\right]\left(\frac{1}{3}TE_{5}+\frac{2}{3}\Delta TE\right)$$

(5) $$m_{c}\left[n,7\right]={\bar{G}}_{c}^{+}\left[n\right]\left(\frac{1}{3}TE_{5}+\frac{4}{3}\Delta TE\right)$$

Moreover, to allow a more effective rephasing, the nonzero value is split into $\left[\frac{1}{5},\frac{2}{5},\frac{3}{5},\frac{4}{5},\frac{5}{5}\right]{\bar{G}}_{c}^{+}\left[n\right]$ or $\left[\frac{1}{5},\frac{2}{5},\frac{3}{5},\frac{4}{5},\frac{5}{5}\right]{\bar{G}}_{c}^{‐}\left[n\right]$ if either ${\bar{G}}_{c}^{+}\left[n\right]$ or ${\bar{G}}_{c}^{‐}\left[n\right]$ is zero. In addition to the inserted z‐shim gradients, for all variants in Figure 1 a navigator echo is acquired after the last echo to compensate for physiologically induced field variations.^33^

### 2.3 $R_{2}^{*}$ estimation

For all measurements, the complex‐valued raw data were first corrected with the phase of the navigator echo as described by Wen et al,^34^ followed by a coil combination using the method proposed by Luo et al.^35^ Then, $F_{z‐shim}\left(TE_{i}\right)$ was calculated as described in Soellradl et al^23^ for the model $F_{4}\left(t\right)$. In this model, ${\underline{M}}_{\mathrm{xy}}\left(z\right)$ is estimated for a certain RF pulse shape and $G_{\mathrm{slice}}$ with a numerical Bloch solver.^36^ Additionally, two potential factors that might affect ${\underline{M}}_{\mathrm{xy}}\left(z\right)$ were included: first, the nominal flip angle deviations due to the transmit RF field $B_{1}^{+}$ and second, $G_{z}$ is superimposed with $G_{\mathrm{slice}}$, which leads to a change of the spatial encoding from z to $z^{\prime }=z\lambda$ with $\lambda =\frac{G_{\mathrm{slice}}}{G_{z}+G_{\mathrm{slice}}}$.^37^ Thus, depending on the sign and amplitude of $G_{z}$, the nominal slice thickness Δz is changed to $\Delta z^{\prime }$, which is given by $\Delta z^{\prime }=\Delta z\lambda$.

After the estimation of ${\underline{M}}_{\mathrm{xy}}\left(z\right)$, $F_{z‐shim}\left(TE_{i}\right)$ was calculated for each echo by substituting $G_{z,input}\left[i\right]$ with $G_{z,input}\left[i\right]=G_{z}+{\bar{G}}_{c}\left[i\right]$ to include the z‐shim gradients. Using $F_{z‐shim}\left(TE_{i}\right)$, $R_{2}^{*}$, and $S_{0}$ were estimated by nonlinear fitting of the reconstructed magnitude data to Equation (2) using the *lsqnonlin()* function in *MATLAB* (MathWorks, Natick, MA).

### 2.4 Sequence and model evaluation

The differences between the investigated sequences and the proposed signal modeling were assessed by calculating four different $R_{2}^{*}$ maps: From the measured data of all three sequences, $R_{2}^{*}$ was estimated with the signal model described previously. Additionally, $R_{2}^{*}$ maps were calculated by fitting the standard spoiled mGRE data to a mono‐exponential signal decay $\left(S_{\mathrm{mono}}\left(TE_{i}\right)=S_{0}e^{‐R_{2}^{*}TE_{i}}\right)$.

### 2.5 Phantom experiments

All experiments were carried out on a whole‐body 3T MRI system (Magnetom Prisma; Siemens, Erlangen, Germany) using an eight‐channel knee coil. To evaluate the proposed z‐shim pattern, a homogenous phantom (5 g/L agar doped with 110 µmol/L Magnevist to shorten T_1_) was built. Measurements with a spoiled 2D mGRE (Figure 1A), a global z‐shim pattern (Figure 1B), and the proposed slice‐specific z‐shimming approach (Figure 1C) were performed. To allow a comparison between the acquisition methods for the estimation of $R_{2}^{*}$, all sequence parameters were set identically—except the amplitudes of the z‐shim gradients. A sinc‐Hanning windowed RF excitation pulse (pulse duration T_pulse_ = 2 ms, time‐bandwidth product = 2.7) with flip angle α = 60° was used. In total, 20 echoes with a monopolar readout and a bandwidth = 500 Hz/Px were acquired. The echo spacing was 3.4 ms for the first four echoes without z‐shim gradients, starting with TE_1_ = 2.8 ms up to TE_4_ = 12.9 ms. For the subsequent echoes with z‐shim gradients (t_c_ = 2 ms), the echo spacing was increased to 5.4 ms (TE_5_ = 18.2 ms to TE_20_ = 98.8 ms). After the 20th echo, phase encoding was rewound to acquire a navigator echo at TE_navi_ = 103.4 ms. A total of 26 slices with a spatial resolution of 1 × 1 × 4 mm^3^ (FOV = 128 × 128 mm^2^) were acquired in an interleaved slice‐acquisition scheme with a TR = 3 seconds. For all z‐shimming approaches, z‐shim gradients were applied with t_c_ = 2 ms starting after the fourth echo. For the measurements with the global z‐shim pattern (Figure 1B), ${\bar{G}}_{c}^{+/‐}$ was set to ±100μT/m. This value was approximately half of the maximum magnitude of the observed field gradients $G_{z}$ in the phantom. In addition to the mGRE sequences, a B_1_ map was acquired using a Bloch‐Siegert approach.^38^

### 2.6 Prescan to estimate ${\bar{G}}_{c}^{+/‐}\left[n\right]$

For the proposed z‐shim approach, a prescan was done to estimate ${\bar{G}}_{c}^{+/‐}\left[n\right]$. The prescan acquisition was performed with the same slice thickness (4 mm), an in‐plane resolution of 2 × 2 mm^2^ (FOV = 64 × 64 mm^2^), three echoes with TE = 2.7 ms, 4.8 ms and 6.9 ms, and GRAPPA acceleration of 2. The phase data of the prescan was then automatically processed to estimate the positive ${\bar{G}}_{c}^{+}\left[n\right]$ and negative ${\bar{G}}_{c}^{‐}\left[n\right]$ for each slice as follows: The phase data were unwrapped using PRELUDE,^39^ and the field map was estimated by dividing the phase difference by the TE difference between the third and first echo. For evaluation, a mask was created by thresholding the magnitude image, which then was eroded with disk elements (radius of 5 pixels) to eliminate outliers close to the border. To estimate the field gradient map $G_{z,pre}$, the gradient in z‐direction of the field map was calculated in regions within the mask and smoothed with a 3D Gaussian filter (kernel size of 1). Then, the $G_{z,pre}$ map was quantized with an interval of 10 µT/m. For each slice, ${\bar{G}}_{c}^{+}\left[n\right]$ was set to the minimum of negative $G_{z,pre}\left[n\right]$ values (${\bar{G}}_{c}^{+}\left[n\right]=\;\text{min}\left(G_{z,pre}\left[n\right]<0\right)$ and for ${\bar{G}}_{c}^{‐}\left[n\right]$ to the maximum of $G_{z,pre}\left[n\right]$ (${\bar{G}}_{c}^{‐}\left[n\right]=\;\text{max}(G_{z,pre}\left[n\right]>0$). Before scanning with the proposed z‐shimming approach, the specific interecho compensation moments were calculated based on the pattern listed in Table 1.

### 2.7 In vivo experiments

The proposed slice‐specific z‐shimming approach (Figure 1C) was evaluated for in vivo $R_{2}^{*}$ mapping by comparing the results with the global approach (Figure 1B) and the approach without z‐shimming (Figure 1A). In total, 3 subjects were scanned on the same 3T MRI system using a 20‐channel head coil. The study was approved by the local ethics committee, and all subjects gave written informed consent. For all experiments, the same RF excitation pulse as in the phantom measurements was used. Sixteen echoes and one navigator echo were acquired with TE_1_ = 3 ms to TE_4_ = 9.7 ms (without z‐shim gradients, echo spacing = 2.2 ms), TE_5_ = 13.9 ms to TE_16_ = 60.6 ms (with z‐shim gradients t_c_ = 2 ms, echo spacing = 4.2 ms), and TE_navi_ = 64.8 ms. Further sequence parameters included a bipolar readout with bandwidth = 500 Hz/Px, TR = 2.5 seconds, and 35 slices with a voxel size of 1 × 1 × 3 mm^3^ (FOV = 256 × 176 mm^2^). As proposed by Nam et al,^31^ the value of ${\bar{G}}_{c}^{+/‐}$ was set to $\pm 220\frac{\mu T}{m}$ for the global approach. The slice‐specific compensation gradients ${\bar{G}}_{c}^{+/‐}\left[n\right]$ were estimated from a prescan as described for the phantom measurements, except that the mask was generated with the brain extraction tool BET, part of FSL.^39^ Sequence parameters of the prescan were 35 slices with a voxel size of 2.3 × 2.3 × 3 mm^3^ (FOV = 96 × 78 mm^2^), three echoes with TE = 2.7 ms, 4.8 ms and 6.9 ms, and a GRAPPA acceleration factor of 3 with 20 reference lines, TR = 344 ms, α = 20°.

The acquisition times were 15 seconds for the prescan and 7 minutes 20 seconds for each of the three GRE sequences. In addition to the mGRE sequences, an MPRAGE sequence with 1‐mm^3^ isotropic resolution was acquired for anatomical segmentation. Furthermore, B_1_ mapping was performed with a highly accelerated approach based on the Bloch‐Siegert shift.^40^

The different methods were compared by calculating the median and interquartile range of $R_{2}^{*}$ values in global white‐matter and gray‐matter masks. The global white‐matter masks were obtained from MPRAGE images using SIENAX,^41^ part of FSL,^39^ and subcortical gray‐matter masks using FSL FIRST.^42^ Regional $R_{2}^{*}$ evaluation (median; interquartile range) was performed after affine registration to mGRE space with FSL FLIRT.^43^, ^44^

## 3 RESULTS

### 3.1 Phantom

**FIGURE 2:**
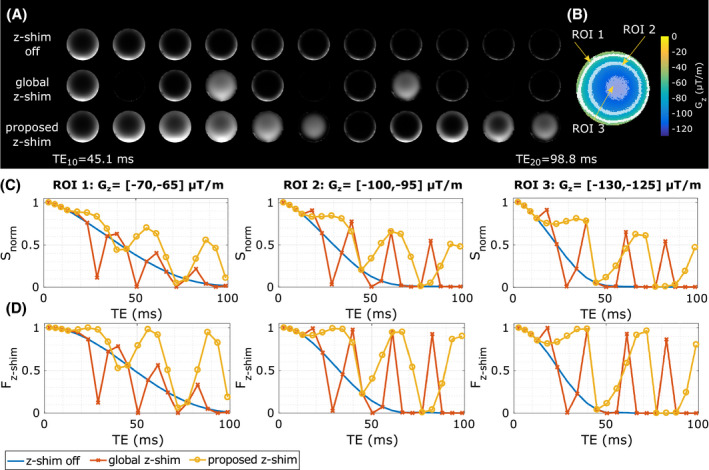
Comparison of the measured averaged normalized signal decay $S_{\mathrm{norm}}\left(TE_{i}\right)=S\left(TE_{i}\right)/S\left(TE_{1}\right)$ and the estimated dephasing functions $F_{z‐shim}\left({\mathrm{TE}}_{i}\right)$ within one slice. A, The magnitude images of $TE_{10}$ to $TE_{20}$. B, Regions of interest (ROIs) were defined within different field gradient intervals, $G_{z}$. In these ROIs, the $S_{\mathrm{norm}}\left(TE_{i}\right)$ (C) and the averaged $F_{z‐shim}\left(TE_{i}\right)$ (D) were estimated. The lines in (C) and (D) show the results from a spoiled mGRE sequence without z‐shim gradients in blue, with the global z‐shim approach ( $|{\bar{G}}_{c}^{+/‐}|=100\mu T/m$) in red, and with the proposed slice‐specific z‐shimming in yellow. Note: The interpolation between echoes is solely for illustration purposes

Figure 2 shows the signal decay of the three investigated pulse sequences within one slice. To demonstrate effects of varying $G_{z}$, three regions of interest (ROIs) (Figure 2B) with different $G_{z}$ intervals were defined, and their normalized averaged signal decay $S_{\mathrm{norm}}$ (Figure 2C) and averaged $F_{z‐shim}$ (Figure 2D) were plotted. The standard spoiled mGRE sequence reveals a faster decay of $S_{\mathrm{norm}}$ with increasing magnitude of $G_{z}$, whereas for the z‐shim approaches, $S_{\mathrm{norm}}$ is differently rephased or dephased. For the global z‐shim, the best signal rephasing is achieved in ROI 2, where ${\bar{G}}_{c}^{+}\approx$ $‐G_{z}=100\mu \frac{T}{m}$, followed by ROI 3. In ROI 1, on the contrary, with an $G_{z}$ interval of $G_{z}=\left[‐70,‐65\right]\mu \frac{T}{m}$, only a small portion of the signal is rephased. In contrast to the global z‐shim, the prescan‐estimated compensation gradients for the proposed approach were ${\bar{G}}_{c}^{+}\left[n=4\right]=125\mu \frac{T}{m}$ and ${\bar{G}}_{c}^{‐}\left[n=4\right]=0$. Thus, only positive compensation gradients were applied in five fractions ($\left[25,50,75,100,125\right]\mu \frac{T}{m}$). Depending on the $G_{z}$ interval of each ROI, the best compensation varies with TE for the proposed approach.

**FIGURE 3:**
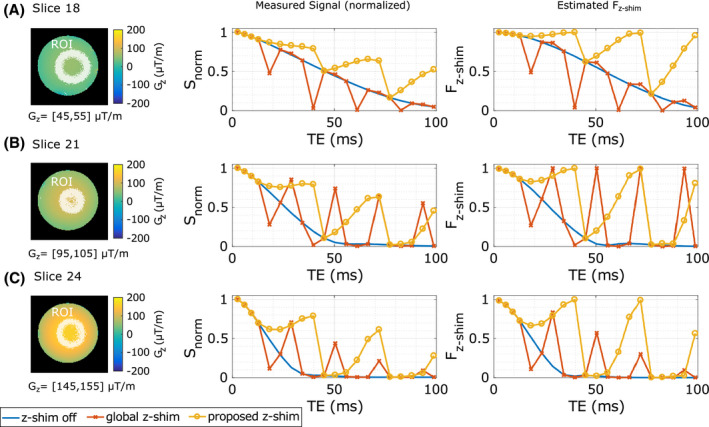
Comparison of the measured averaged normalized $S_{\mathrm{norm}}\left(TE_{i}\right)=S\left(TE_{i}\right)/S\left(TE_{1}\right)$ (middle) and the averaged estimated dephasing functions $F_{z‐shim}\left({\mathrm{TE}}_{i}\right)$ (right) in three slices (A, B, and C). In each slice, averaging was performed in a ROI defined by different intervals of field gradients $G_{z}$ (left). The lines in the plots show the results from a spoiled mGRE sequence without z‐shim gradients in blue, with the global z‐shim approach ( $|{\bar{G}}_{c}^{+/‐}|=100\mu T/m$) in red, and with the proposed slice‐specific z‐shimming in yellow. Note: The interpolation between echoes is solely for illustration purposes

In Figure 3, $S_{\mathrm{norm}}$ and $F_{z‐shim}$ are plotted as a function of the TE for three different slices. In each slice, the values were averaged within ROIs of different $G_{z}$ interval. Similar to Figure 2, with the global approach, the best signal rephasing is achieved when ${\bar{G}}_{c}^{‐}\approx ‐G_{z}\approx ‐100\mu T/m$ (Figure 3B). In contrast, with the proposed approach the signal is gradually rephased for all slices for each block of compensation gradients ( ${\bar{G}}_{c}^{+}\left[n=18,21,24\right]=0)$. Compared with the global approach, the estimated ${\bar{G}}_{c}^{‐}\left[n\right]$ for the depicted slices were ${\bar{G}}_{c}^{‐}\left[18\right]=‐55\mu \frac{T}{m}$, ${\bar{G}}_{c}^{‐}\left[21\right]=‐105\mu \frac{T}{m}$, and ${\bar{G}}_{c}^{‐}\left[24\right]=‐175\mu \frac{T}{m}$, which are close to the range of $G_{z}$ values within the ROIs. Therefore, after each fifth compensation gradient, the signal is nearly ideally compensated in each block. This is indicated when comparing $S_{\mathrm{norm}}$ of the echoes $TE_{9}=39.7ms$ and $TE_{15}=71.9ms$ with $F_{z‐shim}$. Here, the dephasing functions $F_{z‐shim}\approx 1$ suggesting an ideal compensation of $G_{z}$. Furthermore, when comparing $S_{\mathrm{norm}}$ between the slices, $S_{\mathrm{norm}}$ is approximately equal for these echoes independent of $G_{z}$.

**FIGURE 4:**
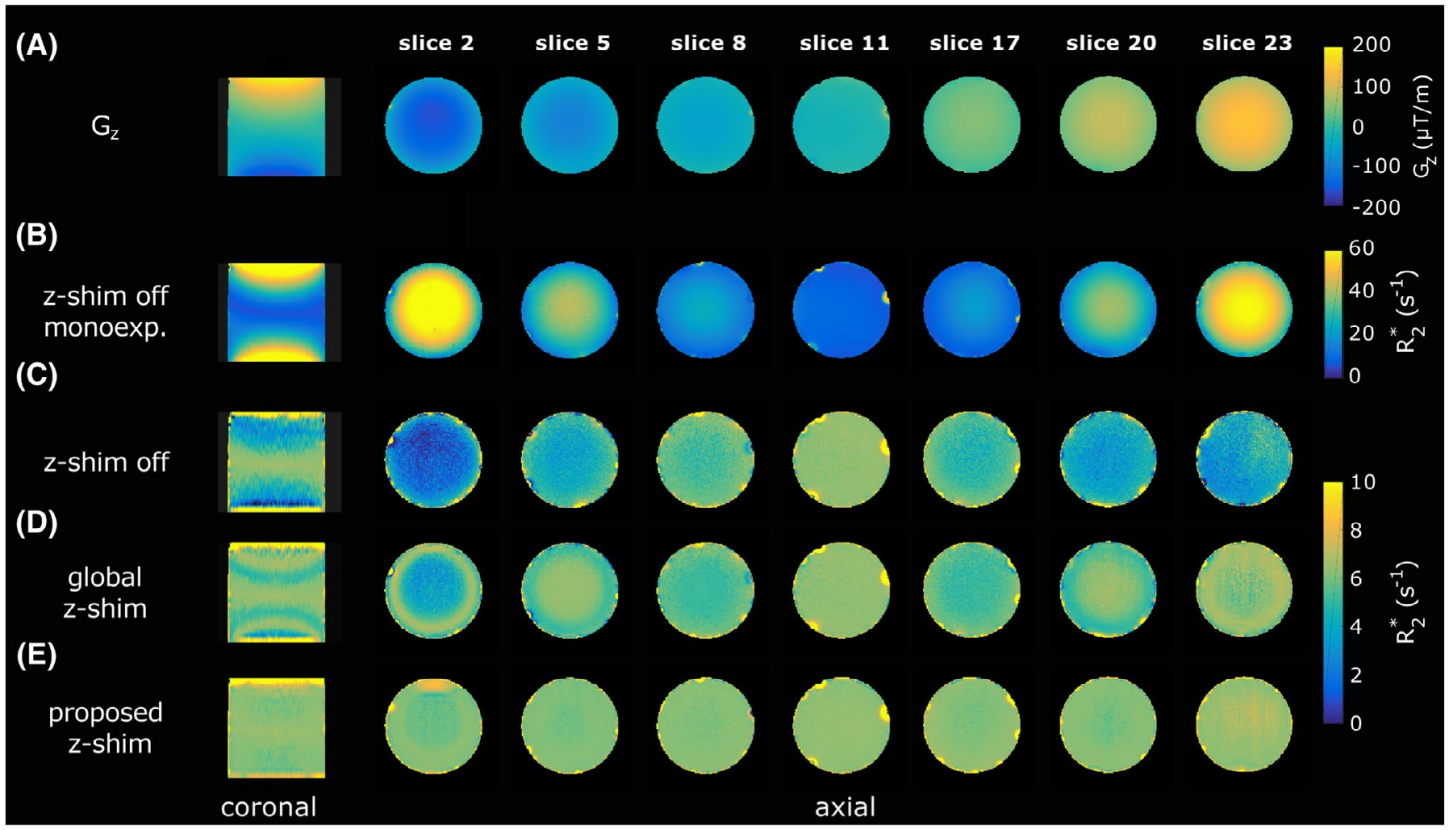
Comparison of estimated $R_{2}^{*}$ maps of a homogenous phantom. A, The field gradient map $G_{z}$. B, The $R_{2}^{*}$ maps were calculated from the spoiled mGRE data by assuming a mono‐exponential signal model neglecting $G_{z}$ ( $F_{z‐shim}=1)$. The other $R_{2}^{*}$ maps were calculated with the proposed signal model using the data of the spoiled mGRE (C), from the global z‐shim ( $|{\bar{G}}_{c}^{+/‐}|=100\mu T/m$) (D), and from the proposed slice‐specific approach (E)

Figure 4 shows the estimated $G_{z}$ map (Figure 4A) and the obtained $R_{2}^{*}$ maps (Figure 4B‐F). The $R_{2}^{*}$ map from the mono‐exponential fit of the standard spoiled mGRE (Figure 4B) reveals a strong overestimation proportional to $\left|G_{z}\right|$, which can be drastically decreased by accounting for $G_{z}$ in the signal model (Figure 4C). Nonetheless, compared with the $R_{2}^{*}$ value of 6.4 s^−1^ in the center of the phantom ( $G_{z}$ is close to zero), $R_{2}^{*}$ becomes underestimated with increasing $\left|G_{z}\right|$. Applying a global z‐shim ( $|{\bar{G}}_{c}^{+/‐}|=100\mu T/m$) improves the results, especially in areas with $\left|G_{z}\right|$ around 100μT/m (Figure 4D, slices 5 and 20). Figure 4E demonstrates that the proposed slice‐specific approach yields more homogenous $R_{2}^{*}$ maps over a wide range of $G_{z}$ values (eg, slices 2 and 23).

**FIGURE 5:**
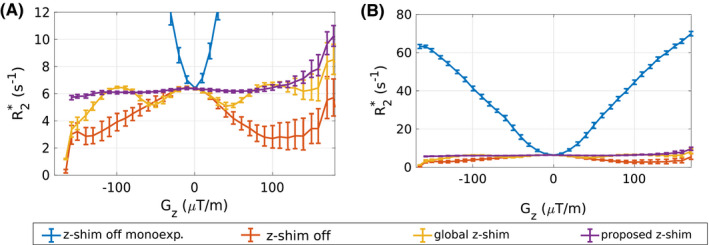
A,B, The $R_{2}^{*}$ values obtained from the phantom experiments as a function of the field gradient $G_{z}$ (bin size = 10 µT/m) with different scaling of the $R_{2}^{*}$ axes . From the spoiled mGRE data, $R_{2}^{*}$ values were first estimated assuming a mono‐exponential signal model (blue line) neglecting $G_{z}$ ( $F_{z‐shim}=1)$, and second by using the proposed model (red line). Furthermore, $R_{2}^{*}$ values from the global z‐shim approach (| ${\bar{G}}_{c}^{+/‐}\left|=100\mu \frac{T}{m}\right)$ (yellow) and the proposed slice‐specific method (purple) are plotted. The $R_{2}^{*}$ values are shown as median and 25^th^ and 75^th^ percentiles (whiskers)

Figure 5 shows the averaged $R_{2}^{*}$ values of the phantom with a bin size of $G_{z}=10\mu \frac{T}{m}$ as a function of $G_{z}$, and demonstrates the difference between the proposed approach and the global z‐shimming. Although the global z‐shim approach ( $|{\bar{G}}_{c}^{+/‐}|=$ $100\mu \frac{T}{m}$) corrects $R_{2}^{*}$ values at about $|G_{z}|=100\mu \frac{T}{m}$ to the expected value of 6.4 s^−1^ ( $R_{2}^{*}$ value at $G_{z}\approx 0\mu \frac{T}{m}$), the proposed approach yields constant $R_{2}^{*}$ values over a broad range of $G_{z}$ from $‐150\mu \frac{T}{m}$ to $125\mu \frac{T}{m}$. Furthermore, the results from the mono‐exponential fit of the standard spoiled mGRE data clearly show the strong increase of $R_{2}^{*}$ with $\left|G_{z}\right|$.

### 3.2 In vivo

**FIGURE 6:**
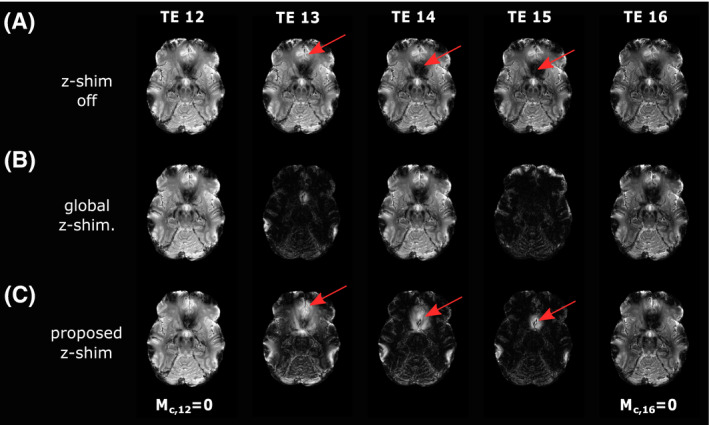
Last five GRE images from TE_12_ to TE_16_ acquired with a spoiled mGRE sequence without z‐shimming (A), with the global z‐shim (B), and with the proposed slice‐specific z‐shimming approach (C). At TE_12_ as well as at TE_16_, the sum of the compensation moments (M_c,12_, M_c,16_) is zero for all sequences. With the proposed approach, the signal can also be rephased in areas where it has already been completely dephased (arrows). The complete series of the echoes with z‐shim gradients is illustrated in Supporting Information Figure S2

Figure 6 shows representative mGRE images for the three investigated sequences (12th to 16th echo). For the spoiled mGRE sequence (Figure 6A), a faster signal decay in areas with strong $G_{z}$, for example, close to the nasal cavities, can be observed. For all sequences, the 12th echo images as well as the 16th echo images are equal because of a zero net moment ( $M_{c,12}=0$ and $M_{c,16}=0$). Between these two echoes, the signal in various brain areas is differently rephased and dephased, depending on the z‐shim approach and $G_{z}$. The global z‐shim pattern with $|{\bar{G}}_{c}^{+/‐}|=220\mu T/m$ shows that negative $G_{z}$ values and positive $G_{z}$ values are rephased at the 13th and 15th, respectively (Figure 6B). Instead of single positive and negative $G_{z}$, a larger range of $G_{z}$ values can be covered by the proposed approach (Figure 6C) (red arrows).

**FIGURE 7:**
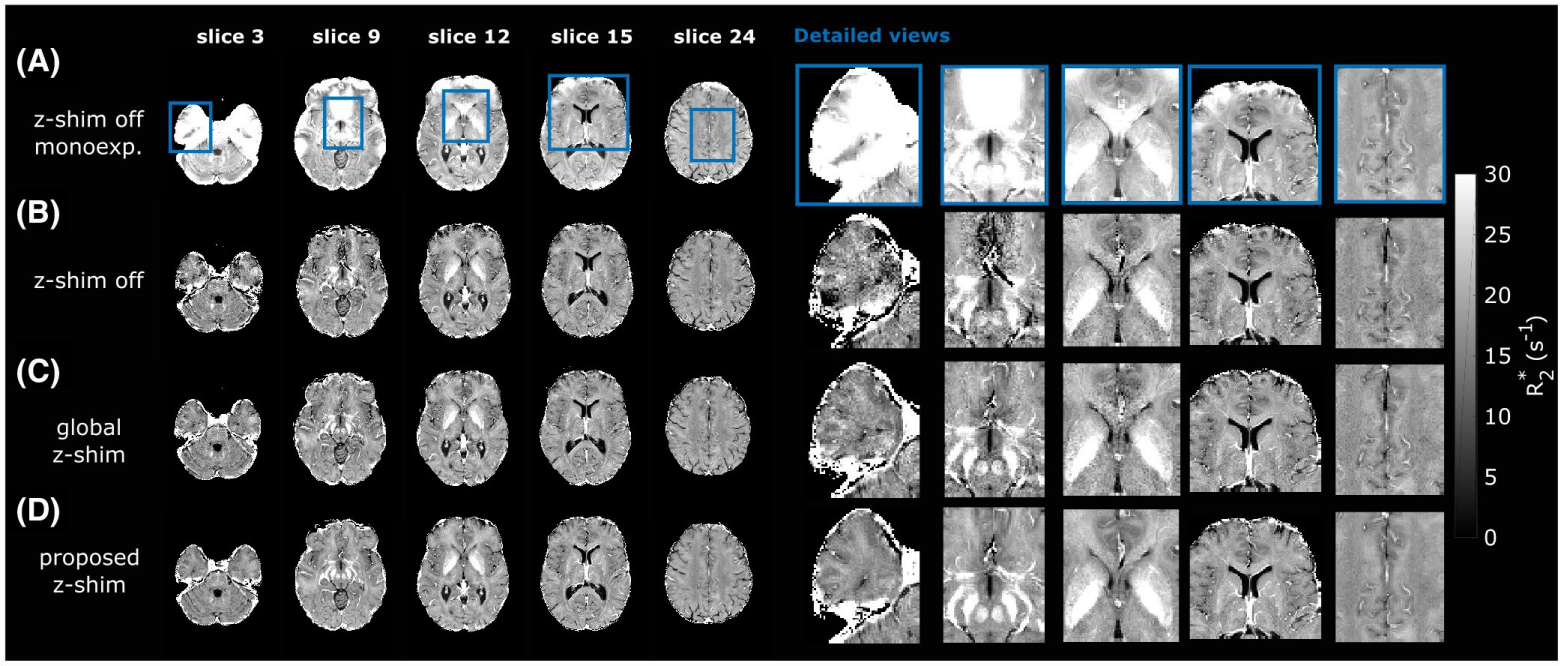
Axial views of estimated in vivo $R_{2}^{*}$ maps (left), with detailed views of the blue rectangular regions (right). A, The $R_{2}^{*}$ maps were directly calculated from the spoiled mGRE data by assuming a mono‐exponential signal model neglecting $G_{z}$ ( $F_{z‐shim}=1)$. The other $R_{2}^{*}$ maps were calculated using the proposed signal model for the spoiled mGRE (B), the global z‐shim ( $|{\bar{G}}_{c}^{+/‐}|=220\mu T/m$) (C), and the proposed slice‐specific approach (D) data. An increase in SNR can be observed from (C) to (D) due to higher signal recovery

The $R_{2}^{*}$ maps in Figure 7 demonstrate improvements in areas with strong $G_{z}$ from the global z‐shim pattern using constant $|{\bar{G}}_{c}^{+/‐}|=220\mu T/m$ (Figure 7C) over the spoiled mGRE (Figure 7A,B), which are most pronounced in the temporal lobe and cerebellum (slice 3) or close to the sinuses (slice 9). Further improvements and additionally increased SNR are observed in the $R_{2}^{*}$ maps obtained with the proposed adaptive z‐shim (Figure 7D).

Table 2 summarizes global and regional $R_{2}^{*}$ values for all subjects. In line with the visual assessment in Figure 7, the interquartile ranges are smallest for the proposed approach in white matter, followed by the global z‐shim method. The largest interquartile ranges were obtained without z‐shimming and when assuming a mono‐exponential signal model.

**TABLE 2:** Regional $R_{2}^{*}\left(s^{‐1}\right)$ obtained with the four evaluated approaches in three subjects

	Method	Global WM	Caudate nucleus	Globus pallidus	Putamen	Thalamus	Brainstem
Subject 1 (m, 33 years)	Z‐shim off monexp.	22.12 (4.26)	20.87 (3.97)	40.34 (8.33)	25.49 (5.11)	23.44 (3.47)	25.91 (6.88)
	Z‐shim off	19.25 (3.31)	19.77 (3.17)	36.03 (8.55)	22.74 (4.06)	19.96 (3.78)	16.99 (7.07)
	Global z‐shim	19.20 (3.19)	19.62 (2.96)	36.05 (8.09)	22.73 (4.16)	19.87 (3.71)	17.07 (5.93)
	Proposed z‐shim	19.20 (2.92)	19.74 (2.95)	35.98 (7.34)	22.77 (3.85)	19.89 (3.29)	17.80 (3.76)
Subject 2 (m, 30 years)	Z‐shim off monexp.	23.74 (4.89)	22.37 (3.93)	38.30 (6.55)	28.55 (5.99)	26.05 (3.66)	25.40 (5.65)
	Z‐shim off	18.75 (3.65)	19.85 (3.31)	31.50 (6.91)	22.33 (4.53)	18.87 (4.89)	17.01 (5.96)
	Global z‐shim	18.81 (3.47)	19.72 (3.09)	31.26 (6.30)	21.97 (4.32)	18.77 (4.30)	16.81 (5.67)
	Proposed z‐shim	18.84 (3.05)	19.78 (2.84)	31.78 (5.75)	22.34 (4.08)	18.93 (3.75)	17.41 (3.96)
Subject 3 (m, 51 years)	Z‐shim off monexp.	22.12 (4.87)	23.88 (5.17)	40.74 (14.08)	29.64 (7.31)	22.95 (4.15)	31.67 (12.46)
	Z‐shim off	19.56 (3.58)	22.35 (3.89)	37.87 (14.98)	27.10 (6.73)	20.60 (3.94)	18.22 (7.87)
	Global z‐shim	19.60 (3.44)	22.12 (3.32)	37.35 (14.27)	27.12 (6.26)	20.53 (3.79)	18.60 (5.81)
	Proposed z‐shim	19.73 (3.22)	22.14 (3.83)	37.38 (13.73)	27.12 (6.18)	20.86 (3.81)	18.69 (4.40)

Note

Data are presented as median (interquartile range).

Abbreviations: GM, gray matter; m, male; monexp., mono‐exponential; WM, white matter.

## 4 DISCUSSION

We have introduced an adaptive slice‐specific z‐shimming approach that allows one to minimize effects of macroscopic field gradients in the slice‐selection direction in 2D mGRE sequences. For each slice *n*, a maximum positive and negative compensation gradient ${\bar{G}}_{c}^{+/‐}\left[n\right]$ is obtained from a fast prescan. To increase the coverage of compensated $G_{z}$ values, ${\bar{G}}_{c}^{+/‐}\left[n\right]$ is split into three fractions: $\left(\left[\frac{1}{3},\frac{2}{3},\frac{3}{3}\right]{\bar{G}}_{c}^{+/‐}\left[n\right]\right)$. Based on these gradient values, a pattern of compensation moments between the echoes is calculated (Figure 1C).

Our novel adaptive slice‐specific z‐shimming was compared with a conventional spoiled mGRE sequence and a global z‐shimming approach that applies a positive and negative ${\bar{G}}_{c}^{+/‐}$ (Figure 1B) independent of the slice position.^31^, ^32^ In contrast to modeling of the standard spoiled mGRE, the global z‐shim enables us to recover $R_{2}^{*}$ values in areas with strong $G_{z}$, which is in line with the results of Nam et al.^31^ By performing slice‐specific z‐shimming with more compensated $G_{z}$ values, the proposed approach results in SNR improvements (Figure 7). Quantitatively, the measured values are within the range of reported values in the literature at 3 T. The z‐shim approach by Nam et al yielded an $R_{2}^{*}$ of $20.77{\;s}^{‐1}$ for the putamen and $34.22{\;s}^{‐1}$ for the globus pallidus,^31^ which is close to the mean values of our 3 subjects with $24.08{\;s}^{‐1}$ and $35.05{\;s}^{‐1}$. When considering the age of the subjects, our $R_{2}^{*}$ values are in good agreement with a study reporting different age ranges.^45^ Subjects’ regional $R_{2}^{*}$ values in the caudate nucleus, thalamus, and brainstem are within the 95% confidence interval of this study.^45^ For subjects 1 and 3, the $R_{2}^{*}$ values in the globus pallidus are slightly above the 95% confidence interval as well as in the putamen for subject 3. For example, in the putamen of subject 3 (51 years), $R_{2}^{*}$ is $27.12{\;s}^{‐1}$ compared with Sedlacik et al, who reported an $R_{2}^{*}$ of $24.3\left(22.1‐26.6\right){\;s}^{‐1}$.^45^

During the optimization process of selecting the optimal ${\bar{G}}_{c}^{+/‐}\left[n\right]$ from the prescan field gradient map $G_{z,pre}\left[n\right]$, splitting of the compensation gradients into different magnitudes was performed. When using a single value (eg, maximum and minimum of positive and negative $G_{z,pre}\left[n\right]$), improvements were only observed in areas with $G_{z}$ values close to the specific compensation gradient. To demonstrate this relation, additional measurements with a slice‐specific approach but with a single ${\bar{G}}_{c}^{+/‐}\left[n\right]$ were performed. As shown in Supporting Information Figure S1, splitting ${\bar{G}}_{c}^{+/‐}\left[n\right]$ led to a more robust compensation over a wide range of $G_{z}$ values. A further refinement of our approach could be made by passing the desired compensation gradient for each echo ${\bar{G}}_{c}^{+/‐}\left[n,TE_{i}\right]$ to the sequence. This comes with the advantage that the compensation gradients can be individually selected, based on the distribution of $G_{z}$ values in each slice.

Z‐shim approaches primarily aim to avoid signal dephasing in areas with large $G_{z}$. In this context, a rather unexpected finding was that also areas with relatively low field gradients (| $G_{z}|<50\mu T/m$) yielded higher SNR in $R_{2}^{*}$ maps by applying small compensation gradients compared with postprocessing‐only methods (Figure 7, slice 24). This SNR increase might be especially promising for combined applications with acceleration methods such as parallel imaging^46^, ^47^, ^48^

The proposed approach has some limitations. First, a prescan with a duration of 15 seconds is necessary to estimate ${\bar{G}}_{c}^{+/‐}\left[n\right]$. However, this additional scan time is minimal compared with the fully sampled z‐shim acquisition (7 minutes 20 seconds) itself, and the increase in SNR compensates for the prolonged scan time. Another issue, especially in vivo, is the estimation of a reliable field gradient $G_{z}$ map from the prescan, which is used to define ${\bar{G}}_{c}^{+/‐}\left[n\right]$. Here, we focused on a robust implementation and avoided potential gradient errors due to missing field‐map values in the skull by eroding the $G_{z}$ map. Nevertheless, it might result in nonoptimal compensation gradients in these areas. An alternative might be to match the slice position to a template $G_{z}$ map instead of performing a prescan.^49^

This work focuses on z‐shimming because the signal dephasing is major along the slice‐selective (z‐)direction compared with the orthogonal directions. In addition, strong in‐plane field gradients can be considered by calculating additional compensation moments in in‐plane directions or, as proposed by Yablonskiy et al,^50^ by modeling the signal dephasing with the voxel spread function.

We have recently introduced a signal modeling approach for an arbitrary excitation pulse and $G_{z}$,^23^ which has been adapted in the current work to describe signal dephasing $F_{z‐shim}$ due to $G_{z}$ and the compensation gradient ${\bar{G}}_{c}$. Because $R_{2}^{*}$ is estimated from the measured data by nonlinear fitting of Equation (2), any modeling error in $F_{z‐shim}$ will propagate into the $R_{2}^{*}$ estimate. Here, $B_{1}^{+}$ and λ have been considered for modeling, but additionally, the ratio $TR/T_{1}$ can affect $F_{z‐shim}$. If the assumption $TR\gg T_{1}$ is not fulfilled, ${\underline{M}}_{\mathrm{xy}}\left(z\right)$ changes according to the steady‐state equation for spoiled gradient‐echo sequences^51^ and might bias $F_{z‐shim}$. To better assess the contributions of $B_{1}^{+}$, λ, and $TR/T_{1}$ to $F_{z‐shim}$, additional simulations were carried out for different $G_{z}$ values (Supporting Information Figure S3). For a ratio of $TR/T_{1}=5$, T_1_ effects are negligibly small, while errors due to $B_{1}^{+}$ increase with α. Compared with $B_{1}^{+}$, the estimated errors caused by λ are similar for each α. In contrast, for $TR/T_{1}=2$, a T_1_ bias can be observed, which is small compared with the $B_{1}^{+}$ error. To investigate the influence of $B_{1}^{+}$ and λ in vivo, Supporting Information Table S1 lists the results without considering $B_{1}^{+}$ and λ. It reveals that the greatest relative change of $R_{2}^{*}$ for the proposed approach was 2.7% for subject 3 in the brain stem (Supporting Information Table S2). These small changes in $R_{2}^{*}$ suggest that B_1_ mapping might not be necessary for the regions evaluated. However, when increasing α or when evaluation regions with stronger $G_{z}$, accounting for $B_{1}^{+}$ might be beneficial. Based on the simulation results, a potential small T_1_ effect cannot be excluded with the TR = 2.5 seconds used in vivo.

Other sources for model deviations in $F_{z‐shim}$ are the input parameters $G_{z}$ and ${\bar{G}}_{c}$. Similar as for the prescan, $G_{z}$ estimation is challenging if the field map values from adjacent slices are missing. For ${\bar{G}}_{c}$, it is assumed that it is ideally characterized by the actual applied gradient moment of the MRI system. Thus, errors might occur in case of gradient imperfections or when a different MRI system is used. In our experiments, a good correspondence between the predicted signal dephasing $F_{z‐shim}$ and the measured signal $S_{\mathrm{norm}}$ (Figures 2 and 3) was observed, indicating a reasonable accurate ${\bar{G}}_{c}$ for the proposed approach.

## 5 CONCLUSIONS

A new adaptive slice‐specific z‐shim approach in combination with signal modeling for 2D mGRE data was introduced to minimize the effects of macroscopic field gradients. The proposed approach allows a more robust correction of $R_{2}^{*}$ maps over a broad range of field gradients and additionally provides a higher SNR.

## Supporting information


**FIGURE S1** Phantom results obtained when extending the global z‐shim pattern (Figure 1B) by a slice‐specific (intermediate) pattern. A, The magnitude images from $TE_{10}$ to $TE_{20}$. B, The $R_{2}^{*}$ maps. The differences of the methods in two regions of interest (ROIs) with different mean $G_{z}$ (C) are assessed by comparing the measured signal decays (D). While with the estimated single ${\bar{G}}_{c}^{‐}\left[n\right]=‐115\mu \frac{T}{m}$, a nearly ideal compensation can be achieved when ${\bar{G}}_{c}^{‐}\left[n\right]\approx ‐G_{z}$ (ROI 1), in the case of heterogeneous $G_{z}$ values, a more robust compensation can be achieved when fractioning ${\bar{G}}_{c}^{‐}\left[n\right]$ (ROI 2). Note: The interpolation between echoes is solely for illustration purposes
**FIGURE S2** Gradient‐echo images from TE_4_ to TE_16_ acquired with a spoiled multi‐echo gradient‐echo (mGRE) sequence without z‐shimming (A), with the global z‐shim (B), and with the proposed slice‐specific z‐shimming approach (C). At TE_4_, TE_8_, TE_12_, and TE_16_, the sum of the compensation moments M_c,4_, M_c,8_, M_c,12_, and M_c,16_ is zero for all sequences. With the proposed approach, the signal can also be rephased in areas where it has been already completely dephased (arrows)
**FIGURE S3** Simulation results for studying the sensitivity of variations in α due to $B_{1}^{+}$, spatial broadening or narrowing of ${\underline{M}}_{\mathrm{xy}}$ with factor $\lambda =\frac{G_{\mathrm{slice}}}{G_{\mathrm{slice}}+G_{z}}$, and incomplete T_1_ relaxation for $R_{2}^{*}$ estimation. The plots show the relative error (%) of $R_{2}^{*}$ as a function of $G_{z}$, estimated from forward simulation of the signal decay with a reference model, which includes $B_{1}^{+}$, λ, and $TR/T_{1}$. While neglecting $TR/T_{1},$ the error was obtained with and without considering $B_{1}^{+}$ and λ for modeling $F_{z‐shim}$ for each parameter combination. In the reference model, the flip angle was scaled with a factor ξ = [0.6, 0.8, 1, 1.2, 1.4] to simulate $B_{1}^{+}$ variations ($\alpha_{\mathrm{sim}}=\alpha \xi$). Then, the spatial coordinates along the slice direction were scaled with λ, and to account for the T_1_ relaxation effects, ${\underline{M}}_{\mathrm{xy}}$ was calculated with the steady‐state equation for spoiled gradient‐echo (GRE) sequences for $TR/T_{1}=2$ and $TR/T_{1}=5$. Simulations were carried out with the same TEs and excitation pulse as used in the in vivo measurements, and $R_{2}^{*}=30s^{‐1}$ was assumed. For $TR/T_{1}=5$, the relative error is negligible when including $B_{1}^{+}$ and λ for all simulated flip angles, because of complete T_1_ relaxation. Thus, T_1_ influence can be neglected. Without $B_{1}^{+}$ and λ, for $\alpha =30^{\circ }$, the error is relatively small and driven primarily by λ. For larger α, the $B_{1}^{+}$ related error increases and becomes the dominant factor. Compared with $\alpha =90^{\circ }$, for $\alpha =60^{\circ }$ the relative error is smaller than 10% over a wide range of $G_{z}$ and ξ. In contrast, for TR/T1=2, substantial errors due to incomplete T_1_ relaxation can be observed in both models
**TABLE S1** Regional $R_{2}^{*}\left(s^{‐1}\right)$ presented as median (interquartile range) obtained with the four evaluated methods in 3 subjects. Note: Values were estimated without including variations of the nominal flip angle due to $B_{1}^{+}$ and spatial broadening or narrowing of ${\underline{M}}_{\mathrm{xy}}$ with λ caused by the supposition of $G_{z}$ and $G_{\mathrm{slice}}$ in the model for Equation (2)
**TABLE S2** Relative change (%) of $R_{2}^{*}\left(s^{‐1}\right)$ values estimated with (Table 2) and without including $B_{1}^{+}$ and λ variations (Supporting Information Table S1) for modeling $F_{z‐shim}$

## Data Availability

The code and data that support the findings of this study are openly available in R2s mapping at https://github.com/neuroimaging‐mug/R2s‐mapping.
