# Supplementary material for: Adaptive slice‐specific z‐shimming for 2D spoiled gradient‐echo sequences
Source: Magn Reson Med. 2020 Sep 10;85(2):818–30. doi: 10.1002/mrm.28468 (PMC7693070; doi:10.1002/mrm.28468)
Supplement: Supplementary file 1 — FIGURE S1 Phantom results obtained when extending the global z‐shim pattern (Figure 1B) by a slice‐specific (intermediate) pattern. A, The magnitude images from TE10 to TE20. B, The R2∗ maps. The differences of the methods in two regions of interest (ROIs) with different mean Gz (C) are assessed by comparing the measured signal decays (D). While with the estimated single G¯c‐n=‐115μTm, a nearly ideal compensation can be achieved when G¯c‐n≈‐Gz (ROI 1), in the case of heterogeneous Gz values, a more robust compensation can be achieved when fractioning G¯c‐n (ROI 2). Note: The interpolation between echoes is solely for illustration purposes FIGURE S2 Gradient‐echo images from TE4 to TE16 acquired with a spoiled multi‐echo gradient‐echo (mGRE) sequence without z‐shimming (A), with the global z‐shim (B), and with the proposed slice‐specific z‐shimming approach (C). At TE4, TE8, TE12, and TE16, the sum of the compensation moments Mc,4, Mc,8, Mc,12, and Mc,16 is zero for all sequences. With the proposed approach, the signal can also be rephased in areas where it has been already completely dephased (arrows) FIGURE S3 Simulation results for studying the sensitivity of variations in α due to B1+, spatial broadening or narrowing of M_xy with factor λ=GsliceGslice+Gz, and incomplete T1 relaxation for R2∗ estimation. The plots show the relative error (%) of R2∗ as a function of Gz, estimated from forward simulation of the signal decay with a reference model, which includes B1+, λ, and TR/T1. While neglecting TR/T1, the error was obtained with and without considering B1+ and λ for modeling Fz‐shim for each parameter combination. In the reference model, the flip angle was scaled with a factor ξ = [0.6, 0.8, 1, 1.2, 1.4] to simulate B1+ variations (αsim=αξ). Then, the spatial coordinates along the slice direction were scaled with λ, and to account for the T1 relaxation effects, M_xy was calculated with the steady‐state equation for spoiled gradient‐echo (GRE) sequences for TR/T1= [file MRM-85-818-s001.docx]

**Supporting information:** **Adaptive slice-specific z-shimming for 2D spoiled gradient-echo sequences**

**Contributions of fractioning** ${\bar{\boldsymbol{G}}}_{\boldsymbol{c}}^{\mathbf{+/-}}\boldsymbol{[n]}$

In the proposed z-shim pattern (Figure 1C) two modifications of the global z-shim (Figure 1B) are introduced. The first one is that a slice-specific averaged compensation $\bar{G}_{c}^{+/-}[n]$ is estimated from the field gradient map $G_{z}$. The second one is that the gradients are split up in 3 factions of positive and negative $[\frac{1}{3},\frac{2}{3},\frac{3}{3}]\bar{G}_{c}^{+/-}[n]$ (or in 5 if one is zero). In order to assess the contribution of fractioning of $\bar{G}_{c}^{+/-}[n]$, additional phantom measurements (using identical sequence parameters as provided in the manuscript) with an intermediate approach between the proposed and global z-shim have been performed. The intermediate approach uses the same pattern as for the global z-shim (Figure 1B) but with slice-specific compensation gradients.

Supporting Information Figure S1 shows the results obtained from a standard mGRE without z-shim, with a global z-shim, with the intermediate approach, and with the proposed z-shim approach for one slice. Figure S1A shows the magnitude echo images in this slice starting from the echo $TE_{5}=18.2 ms$ up to $TE_{20}=98.8 ms$ for the different approaches. Using the forward model for the signal decay $S(t)$ in Equation (1), the $R_{2}^{*}$ maps (Figure S1B) were estimated. In general, all z-shim approaches perform superior compared with the standard mGRE sequence. However, when closely comparing the intermediate z-shim with the proposed slice-specific z-shim, differences can be observed close to the border of the phantom (blue arrows). Here, the $R_{2}^{*}$ values are underestimated in all cases except for the proposed z-shim. This can be explained by comparing the signal decay in ROIs (Figure S1D) with the field gradient map $G_{z}$ (Figure S1C). In ROI 1 the median of $G_{z}=104 \mu\frac{T}{m}$ is close to the estimated $\bar{G}_{c}^{-}\left[ n \right]=-115 \mu T/m$ ($\bar{G}_{c}^{+}\left[ n \right]=0$), which explains especially the good performance of the slice-selective z-shim, since a substantial amount of signal is rephased by the compensation gradients. In contrast, in ROI 2 the median $G_{z}=69 \mu\frac{T}{m}$ is smaller than $\bar{G}_{c}^{-}\left[ n \right]$ and consequently the signal decay differs. The slice-specific approach with a single compensation gradient (intermediate z-shim) rephases a small portion of the signal. However, in the proposed z-shim, the signal is maximally rephased after the third moment in each block (out of 5), which corresponds to ${\frac{3}{5} \bar{G}}_{c}^{-}\left[ n \right]=-\frac{3}{5} 115 \mu\frac{T}{m}=-69 \mu T/m$. Thus, fractioning the compensation gradients $\bar{G}_{c}^{+/-}\left[ n \right]$ is advantageous if a larger range of $G_{z}$ values is present in a slice.


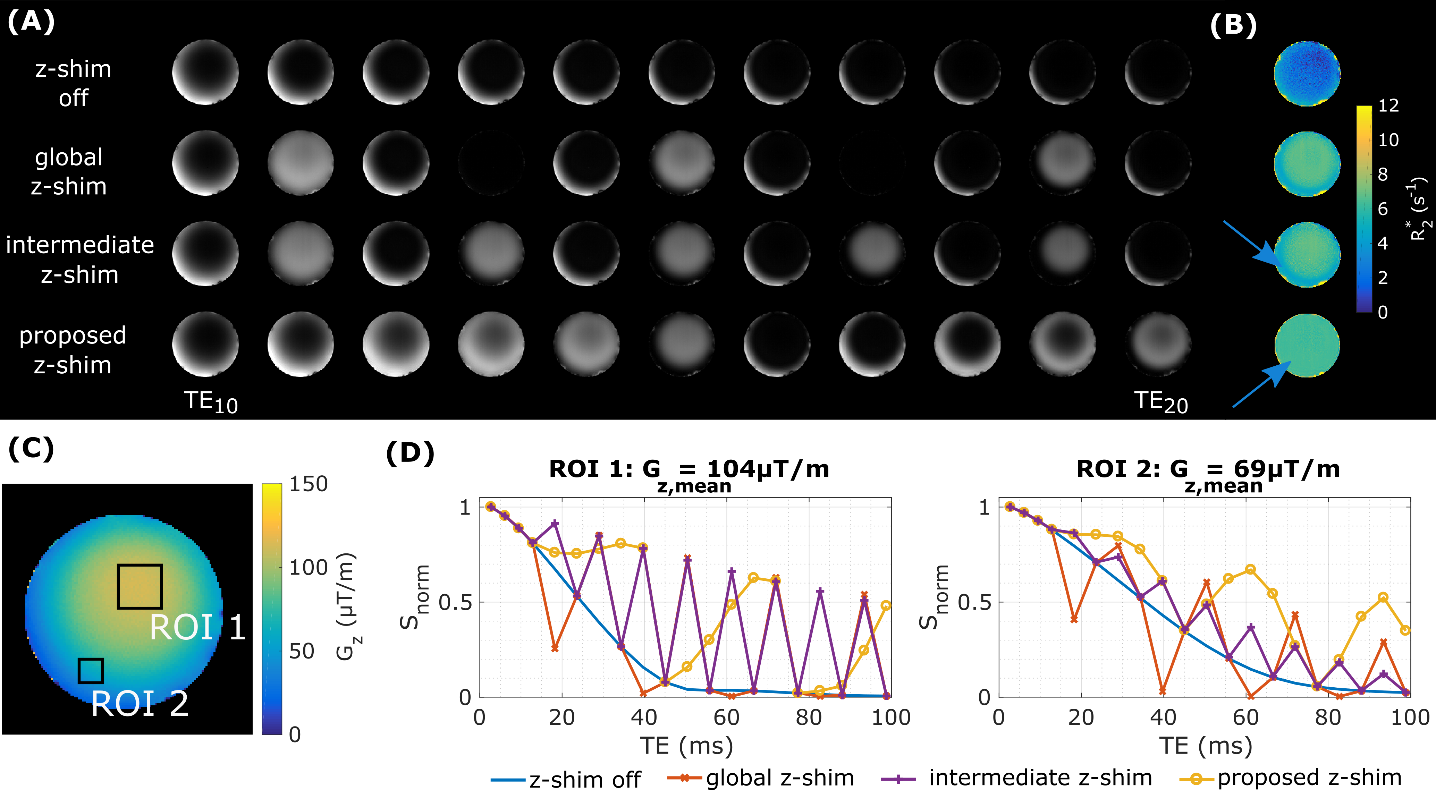


Supporting Information Figure S1: Phantom results obtained when extending the global z-shim pattern (Figure 1B) by a slice-specific (intermediate) pattern. (A) shows the magnitude images from $TE_{10}$ to $TE_{20}$ and (B) the R2* maps. The differences of the methods in two ROIs with different mean $G_{z}$ (C) are assessed by comparing the measured signal decays (D). While with the estimated single $\bar{G}_{c}^{-}\left[ n \right]=-115\mu\frac{T}{m}$ a nearly ideal compensation can be achieved when $\bar{G}_{c}^{-}\left[ n \right]\approx-G_{Z}$ (ROI 1), in case of heterogenous $G_{Z}$ values, a more robust compensation can be achieved when fractioning $\bar{G}_{c}^{-}\left[ n \right]$ (ROI 2). Note: The interpolation between echoes is solely for illustration purpose.


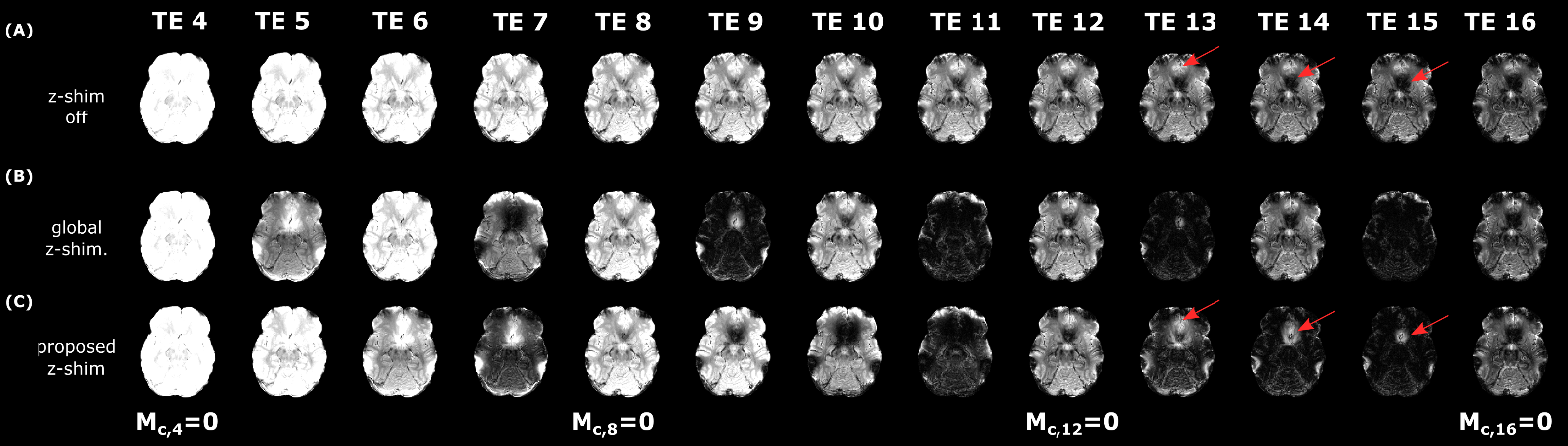


Supporting Information Figure S2: Gradient-echo images from TE_4_ to TE_16_ acquired with a spoiled mGRE sequence without z-shimming (A), with the global z-shim (B), and with the proposed slice-specific z-shimming approach (C). At TE_4_, TE_8_, TE_12_, and TE_16_ the sum of the compensation moments M_c,4_, M_c,8_, M_c,12_, and M_c,16_ is zero for all sequences. With the proposed approach the signal can be rephased also in areas where it has been already completely dephased (arrows).


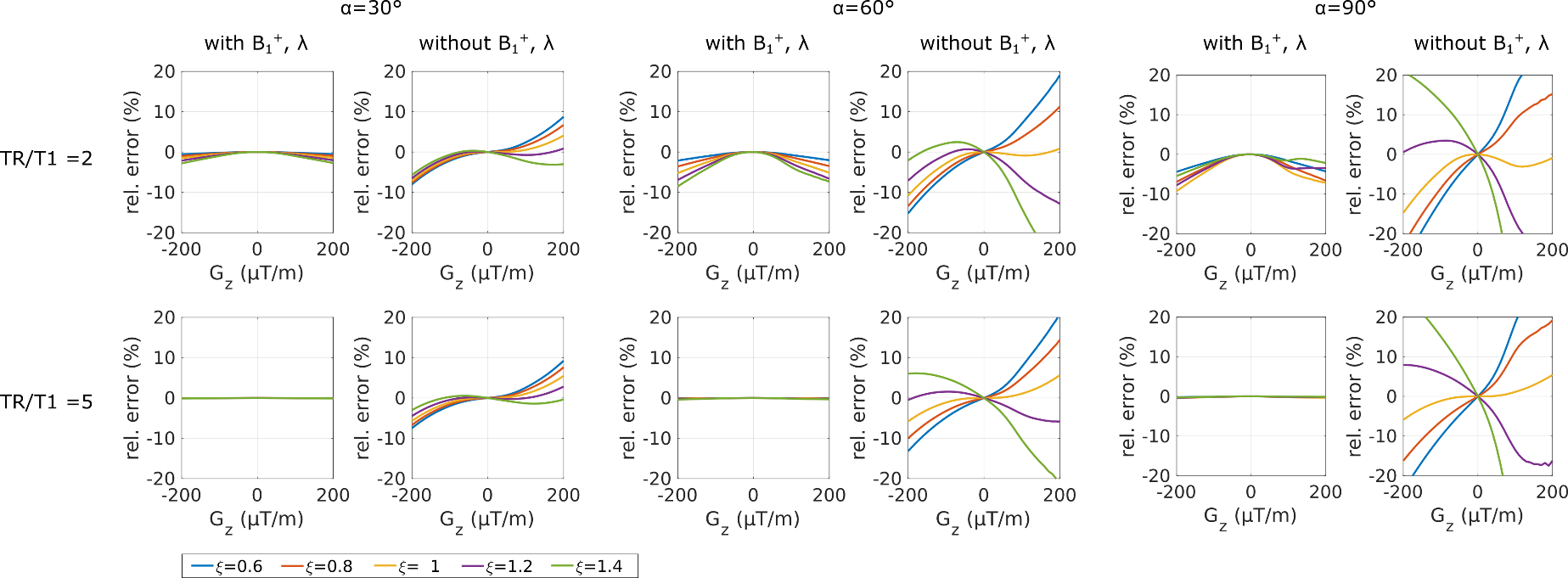


Supporting Information Figure S3: Simulation results for studying the sensitivity of variations in $\alpha$ due to $B_{1}^{+}$, spatial broadening or narrowing of $\underline{M}_{xy}$ with factor $\lambda=\frac{G_{slice}}{G_{slice}+G_{z}}$, and incomplete $T_{1}$ relaxation for $R_{2}^{*}$ estimation. The plots show the relative error (%) of $R_{2}^{*}$ as function of $G_{z}$ estimated from forward simulation of the signal decay with a reference model, which includes $B_{1}^{+}$, $\lambda$, and $TR/T_{1}$. While neglecting $TR/T_{1},$ the error was obtained with and without considering $B_{1}^{+}$ and $\lambda$ for modeling $F_{z-shim}$ for each parameter combination. In the reference model the flip angle was scaled with a factor ξ = [0.6, 0.8, 1, 1.2, 1.4] to simulate $B_{1}^{+}$ variations ($\alpha_{sim}=\alpha\xi$). Then, the spatial coordinates along slice direction were scaled with $\lambda,$ and to account for the $T_{1}$ relaxation effects, $\underline{M}_{xy}$ was calculated with the steady-state equation for spoiled GRE sequences for $TR/T_{1} =2$ and $TR/T_{1} =5$. Simulations were carried out with the same echo times and excitation pulse as used in the in vivo measurements and $R_{2}^{*}=30 s^{-1}$ was assumed. For $TR/T_{1} =5$ the relative error is negligible when including $B_{1}^{+}$ and $\lambda$ for all simulated flip angles because of complete $T_{1}$ relaxation. Thus, $T_{1}$ influence can be neglected. Without $B_{1}^{+}$ and $\lambda$ , for $\alpha=30^{\circ}$, the error is relatively small and mainly driven by $\lambda.$ For larger $\alpha$, the $B_{1}^{+}$ related error increases and becomes the dominant factor. Compared with $\alpha=90^{\circ}$, for $\alpha=60^{\circ}$ the relative error is smaller than 10% over a wide range of $G_{z}$ and $\xi$. In contrast, for $TR/T1 =2$, substantial errors due to incomplete $T_{1}$relaxation can be observed in both models.

Supporting Information Table S1: Regional $R_{2}^{*} \left( s^{-1} \right)$ presented as median (IQR) obtained with the 4 evaluated methods in 3 subjects. Values were estimated without including variations of the nominal flip angle due to $B_{1}^{+}$ and spatial broadening or narrowing of $\underline{M}_{xy}$ with λ caused by the supposition of $G_{z}$ and $G_{slice}$ in the model for Equation (2).

|  | Method | Global WM | | Caudate Nucleus | | Globus Pallidus | | Putamen | | Thalamus | | Brainstem | |
| --- | --- | --- | --- | --- | --- | --- | --- | --- | --- | --- | --- | --- | --- |
| subject 1 (m 33 years) | z-shim off monexp. | 22.12 | (4.26) | 20.87 | (3.97) | 40.34 | (8.33) | 25.49 | (5.11) | 23.44 | (3.47) | 25.91 | (6.88) |
|  | z-shim off | 19.33 | (3.31) | 19.79 | (3.14) | 36.37 | (8.47) | 22.92 | (4.12) | 20.29 | (3.68) | 17.90 | (6.74) |
|  | Gc+/- | 19.27 | (3.18) | 19.61 | (2.94) | 36.32 | (8.05) | 22.88 | (4.19) | 20.14 | (3.66) | 17.75 | (5.78) |
|  | proposed z-shim | 19.24 | (2.92) | 19.73 | (2.94) | 36.22 | (7.33) | 22.89 | (3.93) | 20.11 | (3.29) | 17.97 | (3.81) |
| subject 2 (m 30 years) | z-shim off (monexp) | 23.74 | (4.89) | 22.37 | (3.93) | 38.30 | (6.55) | 28.55 | (5.99) | 26.05 | (3.66) | 25.40 | (5.65) |
|  | z-shim off | 19.01 | (3.63) | 20.03 | (3.36) | 32.22 | (6.76) | 22.79 | (4.50) | 19.66 | (4.76) | 18.02 | (5.68) |
|  | Gc+/- | 19.02 | (3.47) | 19.88 | (3.12) | 31.77 | (6.20) | 22.37 | (4.36) | 19.40 | (4.17) | 17.62 | (5.55) |
|  | proposed z-shim | 18.95 | (3.07) | 19.87 | (2.85) | 32.15 | (5.70) | 22.63 | (4.14) | 19.35 | (3.75) | 17.75 | (4.05) |
| subject 3 (m 51 years) | z-shim off (monexp) | 22.12 | (4.87) | 23.88 | (5.17) | 40.74 | (14.08) | 29.64 | (7.31) | 22.95 | (4.15) | 31.67 | (12.46) |
|  | z-shim off | 19.75 | (3.57) | 22.24 | (3.87) | 38.14 | (14.98) | 27.25 | (6.84) | 20.90 | (3.93) | 19.76 | (7.57) |
|  | Gc+/- | 19.75 | (3.43) | 22.03 | (3.34) | 37.70 | (14.05) | 27.28 | (6.31) | 20.80 | (3.80) | 19.44 | (5.75) |
|  | proposed z-shim | 19.85 | (3.22) | 22.08 | (3.83) | 37.74 | (13.71) | 27.26 | (6.33) | 21.12 | (3.81) | 19.21 | (4.46) |

Supporting Information Table S2: Relative change (%) of $R_{2}^{*} \left( s^{-1} \right)$ values estimated with (Table 2) and without including $B_{1}^{+}$ and $\lambda$ variations (Supporting Information Table S1) for modeling $F_{z-shim}$.

|  | Method | Global WM | Caudate Nucleus | Globus Pallidus | Putamen | Thalamus | Brainstem |
| --- | --- | --- | --- | --- | --- | --- | --- |
| subject 1 (m 33 years) | z-shim off monexp. | 0.00 | 0.00 | 0.00 | 0.00 | 0.00 | 0.00 |
|  | z-shim off | -0.42 | -0.08 | -0.95 | -0.81 | -1.64 | -5.35 |
|  | Gc+/- | -0.41 | 0.05 | -0.76 | -0.66 | -1.34 | -3.96 |
|  | proposed z-shim | -0.24 | 0.03 | -0.66 | -0.50 | -1.12 | -0.92 |
| subject 2 (m 30 years) | z-shim off (monexp) | 0.00 | 0.00 | 0.00 | 0.00 | 0.00 | 0.00 |
|  | z-shim off | -1.37 | -0.92 | -2.27 | -2.10 | -4.19 | -5.93 |
|  | Gc+/- | -1.09 | -0.84 | -1.63 | -1.83 | -3.36 | -4.87 |
|  | proposed z-shim | -0.59 | -0.48 | -1.15 | -1.27 | -2.26 | -1.90 |
| subject 3 (m 51 years) | z-shim off (monexp) | 0.00 | 0.00 | 0.00 | 0.00 | 0.00 | 0.00 |
|  | z-shim off | -0.95 | 0.49 | -0.73 | -0.54 | -1.45 | -8.44 |
|  | Gc+/- | -0.77 | 0.41 | -0.95 | -0.60 | -1.32 | -4.51 |
|  | proposed z-shim | -0.61 | 0.27 | -0.97 | -0.50 | -1.25 | -2.74 |
